# Medico-legal interpretations of gas on postmortem CT: a scoping review

**DOI:** 10.1007/s00414-026-03930-x

**Published:** 2026-07-24

**Authors:** Tobias Gelius, Rasmus Möllby, Jonas Bergdahl, Garyfalia Ampanozi, Alexander Tyr

**Affiliations:** 1 Swedish National Board of Forensic Medicine, Stockholm, Sweden; 2 Swedish National Board of Forensic Medicine, Umeå, Sweden; 3 Laboratory of Forensic Medicine and Toxicology, School of Medicine, Aristotle University of Thessaloniki, Thessaloniki, Greece; 4 Department of Laboratory Medicine, Karolinska Institutet, Stockholm, Sweden

**Keywords:** Radiology, PMCT, Forensic pathology, Forensic imaging, Putrefactive gas, Gas embolism, Vacuum phenomenon, Forensic radiology, Diving fatalities, SPICOT

## Abstract

The use of postmortem computed tomography (PMCT) in forensic investigations has increased during recent years. PMCT differs from clinical CT due to the inevitable manifestation of postmortem changes that varies significantly between cases. However, differentiating between gas with an antemortem origin (e.g. gas embolism) vs. gas with a postmortem (putrefactive gas) origin presents a critical challenge in forensic medicine. This scoping review evaluated how gas distribution patterns on PMCT may aid in determining gas origin. In accordance with PRISMA-ScR guidelines, searches were conducted in PubMed, Web of Science, and CINAHL. 10,133 records were identified, of which 28 studies met the inclusion criteria. Overall, evidence was limited and heterogeneous, with substantial variation in terminology, gas assessment methods and study populations. Evidence suggests that differentiating pathological gas from putrefactive, resuscitation-related artefacts, and postmortem effects such as off-gassing remains a significant diagnostic challenge postmortem. Nevertheless, certain early PMCT patterns may assist interpretation. Within the first 24 h postmortem, intravascular and cardiac gas more commonly reflect systemic or traumatic mechanisms, although putrefaction cannot be excluded. Isolated cardiac gas may result from resuscitation efforts, whereas combined cardiac and intrahepatic gas more strongly suggests decomposition. In diving fatalities, embolic gas findings may be compounded by postmortem off-gassing as well as decomposition changes, and should be interpreted alongside dive profiles and, where available, gas sampling. Small gas collections adjacent to laryngeal structures may indicate trauma when other sources and decomposition are excluded. Overall, integrating PMCT gas distribution with contextual information and ancillary analyses may help understand gas origin, but further targeted research is required.

## Introduction

The use of postmortem (PM) computed tomography (CT) in forensic investigations has increased during recent years as a diagnostic complement to conventional autopsy [1]. PMCT provides forensic pathologists with a unique ability to visualize anatomical structures, injury patterns, localization of foreign bodies, and the distribution of gas throughout the body. Among these, the identification of gas is by far, the defining strength of PMCT, and the implementation technology has facilitated in-depth examination of specific regions, assessment of areas not routinely observed during autopsy, and a different approach to explore pathologies.

Although CT is an established diagnostic modality in clinical medicine, its application in forensic medicine is still less routine [2–4]. Gas detected on CT in both living and deceased individuals may arise from similar underlying mechanisms, including gas embolism, traumatic or iatrogenic disruption of gas-containing organs, and bacterial gas production. However, these findings are often more extensive within the PM setting. In cases where PMCT is performed immediately or shortly after death, the distribution of gas may provide diagnostically relevant information [5], though determining whether the gas has contributed to the cause of death, or merely constitutes an incidental finding (artefactual gas) remains challenging. Moreover, as the PM interval (PMI) progresses, two additional superimposed processes take place, that may obscure diagnostic findings, further complicating image interpretation.

The first process, autolysis, is a non-bacterial enzyme-mediated breakdown of cellular structures that begins immediately upon death, and the second process; putrefaction, is driven by anaerobic bacteria producing gas through reductive metabolism [6]. Bacterial proliferation subsequently disseminates throughout the body via the circulatory system, resulting in widespread gas accumulation. The onset and extent of this process is highly variable and significantly influenced by environmental conditions (such as heat), and individual physiological factors, (for instance body composition or blood loss) [7, 8].

The progressive tissue degradation caused by autolysis, putrefaction and activity of insects, scavengers or larger animals creates secondary spaces occupied by gas. Although putrefactive gas is generally considered to originate within the gastrointestinal wall before extending into the mesenteric and portal venous systems [9, 10], PMCT has enabled more detailed analysis of its distribution, raising the question of whether recognizing such gas patterns may be distinguished from antemortem (AM) gas accumulations. In addition, PM microbial succession also progressively displaces AM pathogens, underscoring the importance of early PMCT and timely microbiological sampling where infectious etiology is suspected [11]. Together, the overlapping processes complicate image interpretation, as determining gas origin and its contribution to the cause of death remains a significant diagnostic challenge.

Here, we define gas embolism, as any gas occurring in the body during physiological circulation, whether produced endogenously or introduced from external sources. Gas can enter both the arterial and the venous system and block either the arterial blood supply of organs (e.g. the brain, leading to serious symptomatology or death), or fill the right cardiac cavities as well as the pulmonary trunk and arteries, leading to right heart failure, respectively. Examples include iatrogenic gas introduced via a catheter (usually venous), decompression illness (DCI) during scuba diving (usually arterial), or gas introduced through open wounds involving large veins with ongoing cardiac activity (venous).

Venous gas embolism typically results from gas entering through vascular injury, travelling to the right heart, and obstructing circulation in the pulmonary arteries or the right cardiac cavities. Gas may also reach the arterial system via a patent foramen ovale or other right-to-left shunts, increasing embolic risk in individuals with pre-existing cardiopulmonary abnormalities [12]. Diagnosing gas embolism as the cause of death is challenging, and traditionally, autopsy techniques employed underwater dissections to identify putative gas in the heart and vascular system. However, this approach is complex, prone to inaccuracies, and only considered in suspected cases. The adoption of PMCT in forensic investigations on the other hand, provides pathologists with superior alternative for detecting intravascular gas [13–16].

In diving-related fatalities, gas may arise from decompression sickness (DCS), caused by in-situ bubble formation from dissolved gas, or arterial gas embolism secondary to pulmonary barotrauma, collectively termed DCI. In pulmonary barotrauma, alveolar gas rapidly expands as pressure decreases, rupturing the alveolar-capillary walls, facilitating the entry of gas bubbles into the arterial circulation and the left cardiac cavities [17]. Pulmonary bullae, present in up to one-third of asymptomatic adults [18], may increase the risk of pneumothorax and/or embolic events. PM off-gassing or PM decompression represents a major diagnostic challenge following diving fatalities, referring to the expansion of dissolved gases within tissues PM that would have been eliminated during ascent via exhalation [19–21]. The resulting widespread gas distribution throughout the body, masks any ability to determine whether gas embolism occurred [19].

Characterizing gas origin in terms of AM or PM using PMCT is challenging since gas exhibits a similar radiographic appearance regardless of its origin. It remains uncertain whether variations in regional distribution patterns, volume, and temporal properties of gases with different origins may permit differentiation based on specific radiological profiles. To answer this, we conducted a scoping review of the existing English-language literature. We aimed to map and synthesize the available evidence on PMCT gas findings and their putative mechanisms and origins. Ultimately, this work seeks to strengthen interpretation, reduce variability, and enhance diagnostic consistency of gas findings in PMCT.

## Methodology

### Research question

A scoping review was designed to answer the primary question: *What evidence exists regarding PMCT gas findings and their putative mechanisms that may assist in differentiating gas origin?* based on criteria established by the Preferred Reporting Items for Systematic Review and Meta-Analysis extension for scoping reviews (PRISMA-ScR) framework [22].

### Search strategy and data sources

Relevant search terms were defined according to our research questions, and categorized into three interventions/blocks to create whole search-strings, as described in Table 1. Search queries were constructed using the Boolean operators “AND” and “OR” and were translated for subsequent searches in the databases of PubMed, Medline, and CINAHL. Records were investigated from inception to 30 April 2024, an updated search was performed in November 2025 to identify any further relevant records published after the initial search. Supplementary sources were also extracted from citations lists of selected studies if deemed relevant.

**Table 1:** Search strategy

Set	Search blocks	PubMed	CINAHL	Web of Science
1	Gas	(“decompression sickness“[tiab] OR “decompression illness“[tiab] OR air[tiab] OR “medical treatment“[tiab] OR Iatrogenic[tiab] OR microbubbles[tiab] OR “air embolism“[tiab] OR “gas embolism“[tiab] OR “oxygen embolism“[tiab] OR gas[tiab] OR barotrauma[tiab] OR hyperbarric[tiab] OR SCUBA[tiab] OR cannulation[tiab] OR “surgical intervention“[tiab] OR “medical intervention“[tiab] OR “off gassing“[tiab] OR “gas collection“[tiab] OR “intravascular gas“[tiab] OR “intraarterial gas“[tiab] OR “intravenous gas“[tiab] OR “cardiac gas“[tiab] OR intrahepatic[tiab] OR “gas distribution”[tiab] intracardial[tiab] OR “pulmonary trauma“[tiab] OR “foramen ovale“[tiab] OR intrasplenic[tiab] OR intrarenal[tiab] OR “intracranial gas“[tiab] OR cerebral[tiab] OR stroke[tiab] OR “compressed air“[tiab] OR “compressed gas“[tiab] OR “gas change”[tiab] OR “gas intoxication“[tiab] OR trauma[tiab] OR “dissolved gas“[tiab] OR diving[tiab] OR “gas poisoning“[tiab] OR “Sepsis“[tiab] OR “Infection“[tiab] OR “systemic air embolism“[tiab] OR “Medical Errors“[Mesh] OR “Gas Poisoning“[Mesh] OR “Microbubbles“[Mesh] OR “Wounds, Penetrating“[Mesh] OR “Barotrauma“[Mesh] OR “Vascular System Injuries“[Mesh] OR “Embolism, Air“[Mesh] OR “Oxygen Inhalation Therapy“[Mesh]) OR (“decomposition“[tiab] OR putrefaction[tiab] OR “Postmortem gas“[tiab] OR decaying[tiab] OR “breaking up“[tiab] OR breakdown[tiab] OR rotting[tiab] OR “gas production“[tiab])	(TI “decompression sickness” OR AB “decompression sickness”) OR (TI “decompression illness” OR AB “decompression illness”) OR (TI air OR AB air) OR (TI “medical treatment” OR AB “medical treatment”) OR (TI iatrogenic OR AB iatrogenic) OR (TI microbubbles OR AB microbubbles) OR (TI “air embolism” OR AB “air embolism”) OR (TI “gas embolism” OR AB “gas embolism”) OR (TI “oxygen embolism” OR AB “oxygen embolism”) OR (TI gas OR AB gas) OR (TI barotrauma OR AB barotrauma) OR (TI hyperbarric OR AB hyperbarric) OR (TI SCUBA OR AB SCUBA) OR (TI cannulation OR AB cannulation) OR (TI “surgical intervention” OR AB “surgical intervention”) OR (TI “medical intervention” OR AB “medical intervention”) OR (TI “off gassing” OR AB “off gassing”) OR (TI “gas collection” OR AB “gas collection”) OR (TI “intravascular gas” OR AB “intravascular gas”) OR (TI “intraarterial gas” OR AB “intraarterial gas”) OR (TI “intravenous gas” OR AB “intravenous gas”) OR (TI “cardiac gas” OR AB “cardiac gas”) OR (TI intrahepatic OR AB intrahepatic) OR (TI intracardial OR AB intracardial) OR (TI “pulmonary trauma” OR AB “pulmonary trauma”) OR (TI “foramen ovale” OR AB “foramen ovale”) OR (TI intrasplenic OR AB intrasplenic) OR (TI intrarenal OR AB intrarenal) OR (TI “intracranial gas” OR AB “intracranial gas”) OR (TI cerebral OR AB cerebral) OR (TI stroke OR AB stroke) OR (TI “compressed air” OR AB “compressed air”) OR (TI “compressed gas” OR AB “compressed gas”) OR (TI “gas intoxication” OR AB “gas intoxication”) OR (TI trauma OR AB trauma) OR (TI “dissolved gas” OR AB “dissolved gas”) OR (TI diving OR AB diving) OR (TI “gas poisoning” OR AB “gas poisoning”) OR (TI sepsis OR AB sepsis) OR (TI infection OR AB infection) OR (TI “systemic air embolism” OR AB “systemic air embolism”) OR (MH “Medical Errors”) OR (MH “Gas Poisoning”) OR (MH “Microbubbles”) OR (MH “Wounds, Penetrating”) OR (MH “Barotrauma”) OR (MH “Vascular System Injuries”) OR (MH “Embolism, Air”) OR (MH “Oxygen Inhalation Therapy”) OR (TI decomposition OR AB decomposition) OR (TI putrefaction OR AB putrefaction) OR (TI “postmortem gas” OR AB “postmortem gas”) OR (TI decaying OR AB decaying) OR (TI “breaking up” OR AB “breaking up”) OR (TI breakdown OR AB breakdown) OR (TI rotting OR AB rotting) OR (TI “gas production” OR AB “gas production”)	TS=(“decompression sickness” OR “decompression illness” OR air OR “medical treatment” OR Iatrogenic OR microbubbles OR “air embolism” OR “gas embolism” OR “oxygen embolism” OR gas OR barotrauma OR hyperbarric OR SCUBA OR cannulation OR “surgical intervention” OR “medical intervention” OR “off gassing” OR “gas collection” OR “intravascular gas” OR “intraarterial gas” OR “intravenous gas” OR “cardiac gas” OR intrahepatic OR intracardial OR “pulmonary trauma” OR “foramen ovale” OR intrasplenic OR intrarenal OR “intracranial gas” OR cerebral OR stroke OR “compressed air” OR “compressed gas” OR “gas intoxication” OR trauma OR “dissolved gas” OR diving OR SCUBA OR “gas poisoning” OR Sepsis OR Infection OR “systemic air embolism” OR “Medical Errors” OR “Gas Poisoning” OR “Microbubbles” OR “Wounds, Penetrating” OR “Barotrauma” OR “Vascular System Injuries” OR “Embolism, Air” OR “Oxygen Inhalation Therapy” OR decomposition OR putrefaction OR “Postmortem gas” OR decaying OR “breaking up” OR breakdown OR rotting OR “gas production”)
	Hits	2,833,916	645,922	7,039,026
2	Postmortem	(“postmortem interval“[tiab] OR PMI[tiab] OR autopsy[tiab] OR cadaver[tiab] OR “post-mortem“[tiab] OR “post mortem“[tiab] OR “postmortem change“[tiab] OR “post-mortem change“[tiab] OR “forensic pathology“[tiab] OR “forensic medicine“[tiab] OR “medico-legal“[tiab] OR “forensic science“[tiab] OR postmortal[tiab] OR “Body Remains“[Mesh] OR “Forensic Medicine“[Mesh])	(TI “postmortem interval” OR AB “postmortem interval”) OR (TI PMI OR AB PMI) OR (TI autopsy OR AB autopsy) OR (TI cadaver OR AB cadaver) OR (TI “post-mortem” OR AB “post-mortem”) OR (TI “post mortem” OR AB “post mortem”) OR (TI “forensic pathology” OR AB “forensic pathology”) OR (TI “forensic medicine” OR AB “forensic medicine”) OR (TI “medico-legal” OR AB “medico-legal”) OR (TI “forensic science” OR AB “forensic science”) OR (TI postmortal OR AB postmortal) OR (MH “Body Remains+”) OR (MH “Forensic Medicine+”)	TS=(“postmortem interval” OR PMI OR autopsy OR cadaver OR “post-mortem” OR “post mortem” OR “forensic pathology” OR “forensic medicine” OR “medico-legal” OR “forensic science” OR postmortal OR “Body Remains” OR “Forensic Medicine”)
	Hits	222,892	30,530	165,584
3	Computed tomography	(“computed tomography“[tiab] OR CT[tiab] OR PMCT[tiab] OR MDCT[tiab] OR MSCT[tiab] OR radiology[tiab] OR “whole-body imaging“[tiab] OR “computer tomography“[tiab] OR “computerized tomography“[tiab] OR tomography[tiab] OR “digital imaging“[tiab] OR “postmortem imaging”[tiab] OR “non-invasive imaging“[tiab] OR “Forensic Imaging“[Mesh] OR “Diagnostic Imaging“[Mesh] OR “Multidetector Computed Tomography“[Mesh])	(TI “computed tomography” OR AB “computed tomography”) OR (TI “CT” OR AB “CT”) OR (TI “PMCT” OR AB “PMCT”) OR (TI radiology OR AB radiology) OR (TI “whole-body imaging” OR AB “whole-body imaging”) OR (TI “computer tomography” OR AB “computer tomography”) OR (TI “computerized tomography” OR AB “computerized tomography”) OR (TI tomography OR AB tomography) OR (TI “digital imaging” OR AB “digital imaging”) OR (TI “non-invasive imaging” OR AB “non-invasive imaging”) OR (MH “Forensic Imaging”) OR (MH “Diagnostic Imaging”) OR (MH “Multidetector Computed Tomography”)	TS=(“computed tomography” OR “CT” OR “PMCT” OR “MDCT” OR “MSCT” OR radiology OR “whole-body imaging” OR “computer tomography” OR “computerized tomography” OR tomography OR “digital imaging” OR “non-invasive imaging” OR “Forensic Imaging” OR “Diagnostic Imaging” OR “Multidetector Computed Tomography)
	Hits	3,340,871	195,510	1,065,064
4	#1 AND #2 AND #3	6,396	506	3,231
	**English filter**	**6**,**345**	**506**	**3**,**231**

### Eligibility criteria

Inclusion and exclusion criteria were defined according to the research question, based on the Population, Concept, and Context (PCC) framework, in accordance with PRISMA-ScR guidelines. For a record to be eligible, the study population needed to consist of deceased individuals who had undergone PMCT, regardless of the cause of death (e.g., traumatic or non-traumatic). The concept of interest was the identification and characterization of gas within the body on PMCT, including any radiological criteria, mechanisms or markers used to consider gas origin. The context included forensic or clinically relevant settings, encompassing studies conducted with or without autopsy as a reference standard.

### Selection of evidence

Inclusion criteria consisted of English language records published in peer-reviewed journals only. Data was imported into Microsoft Excel (Office 2019) for selection and cataloguing according to previous works [23]. Following removal of duplications, records were screened for relevance in a systematic and sequential manner, first by title, next by abstract and lastly in full-text. Screening was conducted independently by two researchers (A.T. and T.G.). Disagreements in scoring were solved during consensus discussions. Only articles pertaining to original research were selected for full-text screening with editorials/commentaries, conferences proceedings, case reports and technical protocols excluded.

### Study evaluation

Although not strictly required for PRISMA-ScR, two authors (A.T. and T.G.) independently assessed the selected records using the SPICOT framework [23] to ensure that included studies met established scientific criteria. SPICOT systematically evaluates scientific evidence and the risk of bias in forensic literature by analysing outlined methodological components, including study design, study population, intervention, control/comparison, outcome, and temporal aspects.

Studies were evaluated in each category to determine the combined level of evidence and risk of bias described previously in Tyr et al. [23]. When variations in score impacted the overall SPICOT classification, consensus discussions were held to decide final score by the two authors (A.T. and, T.G.). Studies that both researchers identified as having scored SPICOT-low (0–9 points) were excluded.

### Data extraction

Data extracted from the included studies are summarized in Table 2. Extracted information encompassed publication type, date, study design, and study classification (retrospective or prospective, and descriptive, correlational, causal-effect, or experimental). Details regarding the study population and control groups were recorded, including sample size and stated causes of death. For each included study, we recorded the etiology as stated by the authors, explicitly classified as trauma or non-trauma. When a study did not clearly specify the etiology of a case, it was coded as “not stated”. We did not infer or reinterpret the etiology based on case characteristics (e.g., type of injury or mechanism) to preserve original reporting. Even if studies employed whole-body imaging, we recorded the specific anatomical regions that were further analysed with respect to gas presence. We defined AM gas as; gas introduced or generated within the body during AM blood circulation and PM gas; gas that enters or develops within the body following the cessation of blood circulation.

**Table 2:** Summary of information extracted from studies

Domain	Criterion
Publication type	Original articles
Publication reliability	Peer-reviewed publication
	Date of the publication
Data sources	Human model
Study design	Retrospective or prospective Descriptive, correlation, causal-effect or experimental
Study population/sample	Representativeness of the population/sample
	Inclusion, and exclusion criteria
	Size of the population/sample
Concept	Gas analysis (assessment strategy and potential quantification) Gas type (antemortem/postmortem)
Context	Forensic investigations
	Trauma (e.g. gunshot, fall from heights etc.) and non-trauma (e.g. septic shock, cardiac arrest etc.) Accident, suicide, homicide, natural
Outcome	Description of interpretation and contribution to cause and manner of death investigation

Since this review seeks to examine and summarize the current state of evidence and proposed diagnostic approaches within the literature, several parameters related to gas identification and analysis were systematically documented. These included the methodological approach, the measurement scale used (continuous, ordinal, or binary), to examine cross-study comparability. Binary and ordinal systems risk obscuring meaningful variation through dichotomization and potentially arbitrary threshold selection, respectively, that in turn may hinder cross study comparisons [24], whereas continuous measures offer greater precision but are rarely feasible in small forensic cohorts. We also considered any weighting or quantification strategies, and the specific organs or anatomical sites assessed. For semi-quantitative or binary methods, the procedural steps or levels applied were also recorded. In addition, the PMI and the primary findings related to the detected gas were extracted.

### Ethical considerations

No studies with living human participants or animals were performed by the authors for the purpose of this study.

## Results

### Study selection

**Fig. 1:**
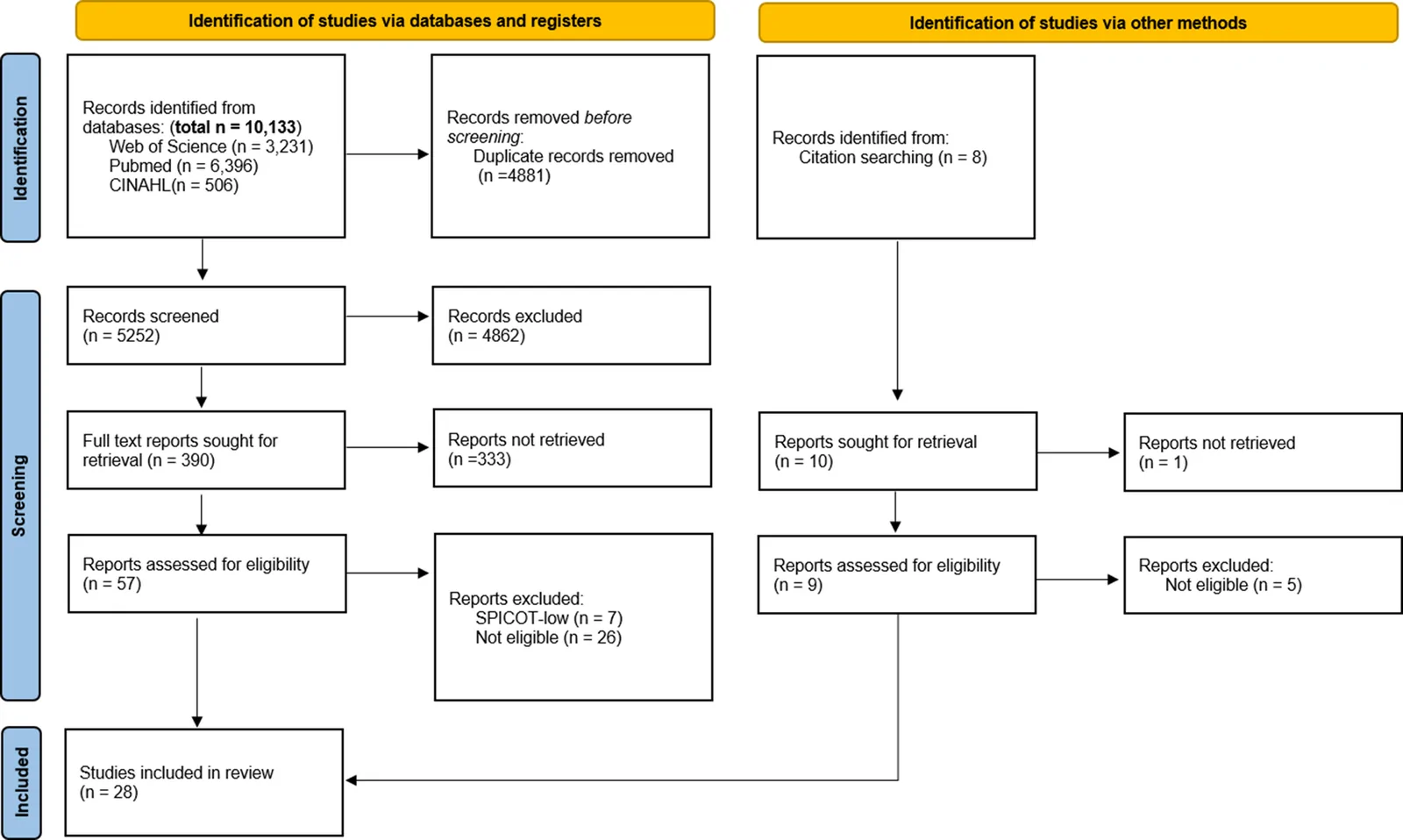
Flowchart detailing study selection

Search results revealed a total of 10 133 records, of which 6 396 were from PubMed, 3 231 from Web of Science, and 506 from CINAHL (Fig. 1). Following removal of duplicates (4 881), and screening of titles and abstracts for relevance, we retrieved a total of 390 full-text articles to be assessed for eligibility. From these, 333 were excluded due to not fulfilling criteria regarding study type, language, peer-review or PCC requirements. Ten additional records were identified from citation searching, with one excluded initially due to lack of relevance and five due to lack of eligibility. The remaining 61 records were further assessed to evaluate the risk of bias. A total of seven records were excluded due to being assessed as SPICOT-low, and 26 were deemed irrelevant upon further examination. A single study was added following an updated search subsequent SPICOT scoring. In total, 28 original studies, published between 2003 and 2025, were eligible for this review.

### Research landscape

The research landscape of the included studies is summarized in Table 3. Studies were classified into two main domains: The first domain considered diagnostic and forensic assessment using gas (*n* = 29), grouped into those studies comprising: trauma and injury assessment (*n* = 17), and PM changes (*n* = 12). The second domain examined methodological and technical evaluation of gas (*n* = 7) and included imaging strengths and limitations (*n* = 5) as well as quantitative indices and validation studies (*n* = 2). It should be noted that a number of studies comprised both domains, and a few studies within the diagnostic and forensic assessment domain considered multiple focus areas.

**Table 3:** Research landscape

Domain	Focus area	References
Diagnostic/forensic assessment	**Injury & disease**	[6, 10, 16, 19, 25–37]
	**Postmortem changes**	[6, 9, 30, 37–45]
Methodology/technical evaluation	**Imaging strengths & limitations**	[29, 33, 44, 46, 47]
	**Quantitative indices & validation**	[30, 43]

*Studies may span over both domains and multiple focus areas

### Study characteristics

A summary of the individual selected study characteristics is shown in Table 4. The majority of studies were of a retrospective study design (*n* = 22; 79%), with the remaining being prospective *n* = 6; 21%). The share between descriptive (*n* = 13; 46%) and correlative (*n* = 15; 53%) studies were more even.

**Table 4:** Characteristics of selected studies

Reference	Primary Aim	Study Design	Population (*n*)	Examined Region	Stated Etiology	Most prominently reported causes of death	PMI
Lopatin et al. 2019 [32]	To evaluate prevalence of pneumothorax on PMCT and correlation with thoracic injuries	Retrospective, correlative	450	Lung	Trauma	Traffic accidents, blunt force, drowning	≤ 2 days
Kudo et al. 2022 [36]	To quantify intervertebral gas on PMCT to differentiate trauma-related separation from other causes	Retrospective, correlative	43	Intervertebral disc	Trauma	Gunshot, blunt/sharp force, traffic accidents, strangulation	-
Smith et al. 2012 [42]	To characterize expected postmortem brain CT findings within 72 h and describe interpretation pitfalls	Retrospective, descriptive	33	Head	Mixed	Penetrating injury other than head, cardiac pathology	≤ 72 h
Guebelin et al. 2021 [45]	To examine correlation of age, sex, and season with decomposition quantified by RA index on PMCT	Retrospective, correlative	440	Multiple regions	Mixed	-	Mean 0.77 days (SD 0.79)
Lyness et al. 2022 [16]	To assess whether PMCT can assist or replace traditional autopsy in cases of suspected suicidal hangings	Retrospective, correlative	50	Neck/throat	-	Hanging	Mean 31 h
Okumura et al. 2022 [10]	To assess PMCT diagnostic accuracy for liver injury in high-energy trauma	Retrospective, Descriptive	97	Liver	Trauma	Traffic accidents, falls from height	< 24 h (*n* = 45); < 25 < 1 week (*n* = 50); ~1 week (*n* = 1)
Oehmichen et al. 2003 [25, 47]	To investigate the cause and significance of intrahepatic gas using PMCT	Retrospective, Descriptive	84	Liver	Mixed	Gunshot, heart failure, strangulation, intoxication, drowning, carbon monoxide, exsangulation, gas embolism	6 h – 4 weeks
Egger et al. 2012 [40]	To investigate the incidence and distribution of gas observed on post-mortem MDCT, and to determine factors that might enable the interpretation gas and vital air embolism	Retrospective, Descriptive	119	Multiple regions	Non-trauma	Hanging; sodium pentobarbital intoxication, other	5–84 h
Fischer et al. 2012 [38]	To assess intrahepatic gas development and radiopacity changes in liver during a 24 h period	Retrospective, Correlative	5	Liver	Non-trauma	-	First scan 6 h PM (average); then hourly for 24 h.
Gebhart et al. 2012 [9]	To evaluate gas presence/distribution on PMCT in non-putrefied trauma and non-trauma cases	Retrospective, Correlative	73	Multiple regions	Mixed	Gunshot, sharp/blunt force, cardiac disease,	Median 16 h
Varlet et al. 2015 [30]	To assess how gas composition analysis supports postmortem imaging in diagnosing causes of death	Prospective, Correlative	40	Multiple regions	Mixed	Drowning, gas embolism, sharp force, fall from height, cardiovascular disease, hanging, intoxication, traffic accident	12 h – 28 years
Laurent et al. 2014 [19]	To describe PMCT appearance of gas collections in fatal diving accidents	Prospective, Correlative	20	Multiple regions	Trauma	Cerebral gas embolism, drowning, cardiac failure	< 72 h
Cartocci et al. 2019 [44]	To evaluate radiological findings and features in advanced decomposed bodies obtained by PMCT with autopsy correlation	Retrospectiv, Descriptive	41	Multiple regions	Mixed	Intoxication, heart failure, sharp force, stangulation, asphyxia, exasanguation, septic chock	4–930 days
Egger et al. 2023 [6]	To describe post-mortem alteration of the vascular system by investigating gas presence and collapsed vessels	Retrospective, Descriptive	118	Heart and vessels	Non-trauma	Natural circumstances, hanging and sodium pentobarbital	3 h to 12 days
Hussein et al. 2021 [37]	To differentiate intraperitoneal decomposition gas vs. pneumoperitoneum on PMCT	Retrospective, Correlative	14	Multiple regions	Non-trauma	Septic peritonitis, hemorrhagic shock	1–7 days
Ishida et al. 2011 [27]	To investigate intravascular gas in abdominal organs in non-traumatic in-hospital deaths	Retrospective, Correlation	50	Abdomen	Non-trauma	Heart, renal and respiratory failure; cancer; esophageal rupture	< 24 h
Giaconi et al. 2022 [31]	To verify differences in gas distribution among diving deaths, fresh and putrefied	Retrospective, Correlation	32	Multiple regions	Mixed	AGE, drowning, other causes of death not defined	24–48 h
Halbertsma et al. 2015 [29]	To study postmortem air-embolism prevalence in neonates after CPR	Prospective, Descriptive	9	Lungs	Non-trauma	-	< 12 h
Borwska-Solonynko et al. 2019 [33]	To evaluate PMCT usefulness in detecting intracranial gas	Retrospective, Descriptive	159	Multiple regions	Trauma	Traffic accidents, fall from heights, blunt/sharp force and gunshot	0–13 days
Chatzaraki et al. 2018 [35]	To investigate the occurrence of the vacuum phenomenon in the intervertebral spaces following complete hanging	Retrospective, descriptive	72	Intervertebral disc	Trauma	Hanging	< 72 h
Shiotani et al. 2004 [26]	To investigate relation between gastrointestinal distension and hepatic portal venous gas on PMCT	Retrospective, correlative	190	Liver	Non-trauma	Cardiac failure, pneumonia,	< 2 h
Shiotani et al. 2010 [34]	To investigate cerebral gas embolism in non-traumatic deaths using PMCT	Prospective, Descriptive	404	Multiple regions	Non-trauma	Fatal hemorrhagic lesions, heart failure	< 2 h
Schulze et al. 2018 [46]	To evaluate the presence of gas in the tissue adjacent to the laryngeal structures in cases of hanging as an indicator of neck trauma	Retrospectiv, correlative	35	Neck/throat	-	Hanging	< 74 h
Egger et al. 2012 [43]	To derive and validate RA-index quantifying radiological alteration using PMCT	Retrospective, Correlative	218	Multiple regions	Mixed	Hanging, pentobarbital intoxication	3 h – 12 days
Cakmak et al. 2025 [41]	To evaluate the relationship between gas and hypostasis detected in organs on PMCT and the PMI	Retrospective, correlative	74	Thoracic and abdominal	-	-	0–5 days
Shiotani et al. 2005 [28]	To investigate cerebral gas embolism on nontraumatic PMCT regarding its frequency, location (arterial or venous), and causes	Prospective, Descriptive	404	Multiple regions	Non-trauma	Fatal hemorrhagic lesions, heart failure	< 2 h
Takahashi et al. 2009 [39]	To investigate the cause and significance of IHG on PMCT.	Retrospective, Descriptive	208	Liver	Non-trauma	-	< ~3 h

The span in total population ranged from five to 450, including studies that incorporated control populations and validation data sets.

In terms of anatomical focus, most studies examined multiple regions (*n* = 12; 43%). Other areas investigated included the head (*n* = 2; 7%), liver (*n* = 5; 17%), lungs (*n* = 2; 7%), intervertebral column (*n* = 2; 7%), neck and throat (*n* = 2; 7%), and the heart (*n* = 1; 3%).

When considering etiology, there was an almost even distribution of studies that focused on trauma-related cases (*n* = 7; 25%), non-trauma cases (*n* = 10; 35%) and those that examined both trauma and non-trauma (denoted as mixed) populations (*n* = 8; 29%). In three studies (10%), the etiology was not stated.

The reported causes of death varied across the studies, with a number of different origins. Traumatic causes typically included gunshot wounds, blunt or sharp force injuries, strangulation, burns, and accidents such as motor vehicle collisions, and falls from heights. Two studies also examined diving incidents and, in some cases, hanging was considered both trauma and non-trauma. Non-traumatic causes encompassed natural diseases, typically sudden cardiac events, organ failure and general hospital-related medical complications. One study considered neonatal deaths. Several investigations examined multiple concurrent causes, such as trauma combined with intoxication, asphyxia, or decomposition, highlighting the wide spectrum of mortality contexts represented in the literature.

Although the reported PMI varied across the reviewed studies, the majority (*n* = 21; 72%) investigated the early PM period, restricted to within one week PM, with frequent cutoffs reported at < 12 h, < 24 h, 24–48 h, and ≤ 72 h. A second cluster of studies (*n* = 3; 10%) examined an extended PMI from approximately one week to 1 year, and two studies (7%) considered a PMI > one year. In two studies (7%) the PMIs not directly stated.

### Gas analysis strategies

To further elucidate how gas analysis had been conducted within the literature, we considered analysis strategies (Table 5). The gas analysis strategies were initially classified according to the methodological approach and further categorized by measurement scale, as either continuous (*n* = 1; 3%), ordinal (*n* = 16; 57%) or binary (*n* = 8; 29%). In a single study (3%) both an ordinal and binary system was employed, and in three studies (7%), no gas assessment strategy was reported. The only study that considered continuous measurement scales employed volumetric analysis following reconstructed 3D images of the liver.

**Table 5:** Gas analysis strategies

Method	Organs/sites assessed	Weighting/approach	Measurement scale	Steps/levels	References
Gas volume	Liver	Quantitative analysis of gas volume in whole liver using reconstructed 3D-renderings	Continuous	-	[25]
Radiological Alteration (RA) index	Heart cavities, liver parenchyma and vessels, left innominate vein, vena cava, abdominal aorta, kidney parenchyma, vertebrate L3 and subcutaneous pectoral tissues	Semi-quantitative visual grading; weighted scoring for postmortem vs. vital embolism	Ordinal	0-III, generating RA index (0–100)	[6, 30, 37, 40, 43–45]
Organ/region-specific scales	Liver, kidney, spleen, pancreas, bowel	Semi-quantitative; gas, vessel or anatomical location/distribution analyzed	Ordinal	3–4 step grading	[25–27, 38, 39, 41]
Anatomical mapping	Intravertebral discs	Semi-quantitative visual grading; anatomical position of gas in sagittal and coronal planes	Ordinal	Trisection on the coronal and sagittal planes, where positions were defined in either grid or distance from landmark	[35, 36]
Quantitative and qualitative scale	Multiple	Semi-quantitative scales adopted to evaluate the radiological findings, alongside the corresponding qualitative scale	Ordinal	8 point visual scale (-- - ++++) with 8 point numeric scale, corresponding to 4 grade qualitatitive scale (absent – high)	[31]
Binary and semi-quantitative scale	Brain	Binary for intravascular and intervertebral gas (IVG); semi-quantitative for pneumocephalus (PNC) and anatomical arrangement	Binary + Ordinal	Presence/absence; and I-III PNC scale.	[33]
Binary quantitative	Multiple	HU density threshold confirms gas presence	Binary	Presence/absence	[9]
Visual/consensus discussions	Multiple	Examiner judgment; descriptive only	Binary	Presence/absence or over/under cut-off	[10, 19, 28, 32, 34, 42, 46]
Not stated	–	–	–	–	[16, 29, 47]

* Studies may span multiple analysis strategies

In terms of ordinal analysis strategies, seven studies (25%) employed the Radiological Alteration (RA) index, a semi-quantitative approach that evaluates gas using a four-step visual grading at seven anatomical sites (heart cavities, liver parenchyma and vessels, left innominate vein, abdominal aorta, kidney parenchyma, vertebrate L3 and subcutaneous pectoral tissues) to determine a weighted scoring between 0 and 100 for the purposes of elucidating its origin.

Six studies (21%) employed organ-specific ordinal scoring systems based on the liver, kidneys, spleen, and pancreas. These assessments were based on semi-quantitative approaches, typically using 3- to 4-step grading scales. Two studies (7%) considered an anatomical mapping strategy, whereby grid patterns were generated on different anatomical planes or distance from landmarks were considered. A single study (3%) included a binary quantitative approach based on HU density to confirm gas in multiple anatomical sites. A combined binary and semi-quantitative scale to evaluate intracranial gas, where intravascular gas was recorded as present or absent, and pneumocephalus was graded using a three-step scale in a single study. Visual assessment and consensus discussions were reported in seven studies (25%), In three studies (10%), no details were provided regarding the methodology used for gas analysis.

### PMCT gas findings

Across the anatomical regions examined, PMCT gas findings demonstrated considerable variability (Table 6). Intrahepatic gas (IHG) findings were largely non-specific, occurring across both traumatic and non-traumatic cohorts independent of cardiopulmonary resuscitation (CPR) status [25, 38, 39]. Distribution pattern on the other hand carried diagnostic value: extravascular focal gas favored high-energy traumatic injury, while diffuse parenchymal gas was more consistent with decomposition [10]. Hepatic portal venous gas (HPVG), visible up 2 h PM, was frequently linked to CPR-related gastrointestinal distension in non-traumatic deaths [26]. Gas in the spleen and pancreas during early PMI most likely reflected decomposition, whereas renal gas remained ambiguous given overlapping contributions from both decomposition and CPR [27].

**Table 6:** Summary of PMCT gas findings by anatomical region

Region	Interpretation of gas finding and origin (where possible)	References
Abdomen		
IHG	Intravascular gas within the hepatic venous system observed during early PMI appears non-specific, occurring in both traumatic and non-traumatic cohorts [25; 26; 27]. Nevertheless, gas embolism is reported to have a stronger association with trauma [25]. Extravascular (focal and surface) gas is more specific for high-energy traumatic liver injury, whereas diffuse parenchymal gas is more consistent with decomposition [10]. The distribution of gas was not correlated with the time elapsed since death, both in cases with and without positive CPR [28].	[10, 25, 27, 28, 38–40]
HPVG	Hepatic portal venous gas may be visible up to 2 h PM and is frequently associated with gastrointestinal distension following cardiopulmonary resuscitation (CPR) in non-traumatic deaths.	[26, 39]
Spleen, pancreas and kidney	Early detection of splenic gas occurrence most likely reflects decomposition patterns in no-CPR cases. Similarly, Pancreatic gas during the initial PMI appears to be rare, and more likely to stem from decomposition than CPR-related air entry. Kidney gas is ambiguous, potentially influenced by both CPR-related mechanisms and decomposition.	[27]
Cardiovascular		
Heart (general)	The main cause of cardiovascular gas in non-traumatic deaths 2 h PM is CPR [30; 31]. Gas appeared in the heart cavities before it appeared in the parenchyma, which may be confused with an air embolism in the early stages of putrefaction Egger et al. [40]. Compositional gas analysis indicates that the presence of H₂, H₂S, and CH₄ is consistent with decomposition. A CO₂/N₂ ratio < 0.2 is associated with gas embolism, whereas a ratio > 0.2 suggests decomposition [30].	[28–30, 34, 40]
Right heart cavities	At an early PM stage, putrefactive gas is preferentially observed in right heart cavities and liver in non-traumatic deaths [32; 33]. On the other hand, complete pneumatization of right ventricle within 24 h PM is consistent with for DCI following diving fatalities [19].	[19, 40, 41]
Left heart cavities	Gas detected in the left heart or periumbilical subcutaneous tissue at an early PMI is inconsistent with a putrefactive [40]. Complete pneumatization of left ventricle within 24 h PM is consistent with for DCI following diving fatalities [19].	[19, 40]
Supraaortic trunk (left ventricle, aortic cross, including the Willis polygon)	Complete pneumatization of supraaortic trunks was best PMCT criterion for DCI following diving fatalities [19]. Gas in left chambers and supraaortic trunks distinguishes AGE divers from fresh and putrefied non-diving corpses even at 24–48 h PM [30].	[19, 30, 31]
Vessels (arterial and venous)	Putrefactive intravascular gas increases with PMI [9; 33; 44]. Vascular collapse likelihood doubled when vessels contain gas [6]. Intravascular gas in upper abdomen within 24 h associated with non-traumatic in-hospital deaths [27].	[6, 9, 27, 41, 43]
Respiratory		
Lungs/pleural space	Pneumothorax is not specific for DCI in diving deaths [19]. No statistically significant link between pneumothorax and death by DCI [19]. Pneumothorax significantly correlated with thoracic injuries in trauma; its presence alone insufficient to infer mechanism [32].	[19, 32]
Head, neck and spine		
Intracranial	PM changes occurring on CT may mimic AM pathology within 72 h [42]. Intracranial gas in traumatic deaths requires careful contextualisation; medico-legal significance varies by distribution and mechanism [33]. Cerebral gas embolism detected on PMCT in non-traumatic cases demonstrate no association with CPR [34].	[33, 34, 42]
Larynx/neck soft tissue	Gas bubble sign adjacent to laryngeal structures in cases of hanging, and may be present without fractures. However, not a reliable standalone marker for trauma.	[16, 46]
Intervertebral discs (thoracic/lumbar)	Central VP in thoracic/lumbar discs associated with hanging in younger individuals; peripheral VP more common in non-trauma controls. Cervical central disc VP may reflect degeneration rather than trauma; interpretation depends on anatomical level and age.	[35, 36]
Multi-regional		
Penetrating wound tracts	Gas accumulations delineate wound tracts in penetrating trauma (gunshot, stabbing), with diagnostic value for trajectory reconstruction.	[9, 47]
RA-index	RA index < 50, with absence of early decomposition features (mesenteric microbubbles, intestinal wall gas), supports antemortem origin of pneumoperitoneum; PMCT within 1 week has limited discriminatory capacity; contrast-enhanced CT recommended for localization of perforation [37]. Combined GEP and RAI show strong correlation between external and internal decomposition and are useful for assessing advanced putrefaction [44]. RAI used as a proxy for decomposition state in PMCT; no significant seasonal effect observed in an urban (mostly indoor) population; significant age and sex differences noted, with higher RAI in older males compared to females, potentially reflecting differences in social isolation [45].	[37, 43–45]

*AM* antemortem, *AGE* arterial gas embolism, *CAGE* cerebral arterial gas embolism, *CPR* cardiopulmonary resuscitation, *DCI* decompression illness, *IHG* intrahepatic gas, *IHVG* intrahepatic venous gas, *HPVG* hepatic portal venous gas, *PMI* postmortem interval, *RA-index* radiological alteration index, *VP* vacuum phenomenon, *GEP* grade of external putrefaction. Origin labels reflect proposed associations in source studies, not established causal relationships. All findings are observational; no causal inference is warranted from correlational data. Several studies appear across multiple rows

In non-traumatic deaths where CPR had been performed, it was considered the predominant cause of cardiovascular gas within two h PM [28, 29]. Generally, putrefactive gas was observed to appear in heart cavities prior to the cardiac parenchyma [40, 41], which may mimic air embolism during the early PMI, though compositional analysis may help distinguish between this. H₂, H₂S, and CH₄ together, indicate decomposition, while a CO₂/N₂ ratio < 0.2 suggests gas embolism and > 0.2 decomposition [30]. Crucially, putrefactive gas preferentially affects the right heart cavities and liver, meaning left heart gas at early PMI is inconsistent with a putrefactive origin [40].

For diving fatalities, complete pneumatization of the left ventricle and supraaortic trunks within 24 h was found to be the strongest PMCT criterion for DCI, remaining discriminatory even at 24–48 h PM (Table 7) [19, 31]. Pneumothorax lacked specificity across multiple contexts: it showed no statistically significant association with DCI in diving fatalities [19], correlated with thoracic injury in trauma but was insufficient to infer a mechanism [32].

**Table 7:** Comparison of PMCT gas findings in diving-related deaths versus non-diving comparators

	Laurent et al. [19]			Giaconi et al. [31]		
Spatial distribution of gas	CAGE divers *n*=7 (PMI = 12 h)	Non-DCI divers*n*=5 (PMI = 16.5 h)	Non-DCI divers *n*= 6 (PMI = 25 h)	AGE divers*n*=10 (PMI 24–48 h)	Fresh corpses*n*=12 (5 drowned/ 7 OCD) (PMI <48 h)	Putrefied corpses*n*=10 (7 drowned / 3 OCD) (PMI > 7 days)
Intravascular / intraarterial gas	100%	54.5%	20%	-	-	-
Venous gas (brachiocephalic, Giaconi et al.)	100%	100%	90%	50%	20% / 14%	100% / 66%
Supraaortic trunks				80%	40% / 14%	100% / 66%
	100%	27.3%	0%			
Left ventricle				80%	0% / 14%	86% / 100%
Right ventricle	100%	36.4%	0%	80%	20% / 29%	100% / 100%
Subcutaneous emphysema limited to thoracic area	57.1%	9.1%	0%	30%	0% / 0%	14% / 0%
Pneumothorax	0%	27.3%	20%	10%	0% / 10%	86% / 100 %

*OCD* other cause of death, *AGE* arterial gas embolism, *CAGE* cerebral arterial gas embolism, *DCI* decompression illness, *PMI* postmortem interval

Intracranial gas findings required careful contextualization, as PMCT changes can mimic AM pathology within 72 h [42], and medico-legal significance varied by distribution and mechanism [33]. Notably, cerebral gas embolism in non-traumatic cases showed no association with CPR [34]. In the neck, a “gas bubble sign” adjacent to laryngeal structures was associated with hanging but could occur without fractures [16, 35, 46]. In the spine, central vacuum phenomenon (VP) in thoracic and lumbar discs was associated with hanging in younger individuals, while peripheral distribution was more common in non-trauma controls; cervical central disc VP was more likely to reflect degeneration, with interpretation dependent on anatomical level and age [35, 36].

It is well established that gas accumulations along wound tracts carry diagnostic value for trajectory reconstruction in penetrating trauma [9, 47]. The RA-index serves as a useful proxy for decomposition state and pathology [43]: an RA-index below 50 with absence of early decomposition features supported an AM origin of pneumoperitoneum, though PMCT within one week had limited discriminatory capacity and contrast-enhanced CT was recommended for localization of perforation [37]. Combined with the grade of external putrefaction (GEP) scheme, the RA-index strongly correlated with external decomposition markers and was useful for staging advanced putrefaction [44]. RA-index showed no significant seasonal variation in urban indoor populations, but was significantly higher in older males than females [45].

## Discussion

PMCT has gained prominence as a preferred imaging modality in forensic medicine, compared to magnetic resonance imaging due to its speed, relative ease of application, and lower cost [40]. In contrast to traditional autopsy, PMCT enables an enhanced capacity to detect and characterize gas, a property that may hold important diagnostic implications [13, 14].

This scoping review sought to map and synthesize the available literature on PMCT gas findings and examine their putative mechanisms and origins. Twenty-eight studies met the inclusion criteria, though few addressed gas origin as a primary aim; the exception being Egger et al. [21, 46], who developed a scoring index specifically to estimate gas origin PM. The remaining literature approached our question indirectly, through investigations of specific mechanisms, anatomical distributions, and temporal patterns across heterogeneous contexts.

The selected studies collectively outline a research landscape spanning two main domains: diagnostic and forensic applications, encompassing trauma assessment, PM alterations, and population data; and methodological development, including imaging performance and validation of quantitative indices. Several studies bridged both domains, underscoring the interdisciplinary and unstandardized nature of PMCT gas analysis. No single PMCT approach currently provides definitive differentiation between AM and PM gas, though certain analytical strategies may offer valuable diagnostic insights into origin.

Initial studies investigating hepatic gas using PMCT sought to determine whether its temporal and spatial distribution could provide insight into its origin. AM gas, such as vital HPVG is a rare condition often associated with mesenteric ischemia, Crohn’s disease or septicemia [48, 49]. Similar gas patterns have been reported on PMCT, where HPVG may be visible PM, and is frequently associated with gastrointestinal distension following CPR in non-traumatic deaths [26, 39]. Shiotani et al. [28] proposed three possible mechanisms of gas formation during CPR: through venous catheterization, pneumatization of dissolved gas through sudden chest compression-decompression and pulmonary injury because of chest compressions [28].

Subsequent forensic studies have demonstrated that IHG is frequently present in both traumatic and non-traumatic deaths, with a predominant localization within the hepatic venous system. Jackowski et al. [25] investigated IHG in traumatic and non-traumatic deaths. Among the 79 non-putrefied corpses, IHG was present in 42 cases (53%), most frequently located within the hepatic venous system. A strong association was observed between IHG and systemic gas embolism (93%), which in turn was more common in traumatic deaths. No significant association was found with PMI (mean 29 h) or gastrointestinal distension, except in cases of severe distension. Overall, Jackowski et al. [25] concluded that IHG in non-putrefied corpses most commonly reflects traumatic gas embolism rather than decomposition or enteric gas entry.

Fischer et al. [38], performed serial CT examinations over 24 h in five non-traumatic deaths without CPR, observing IHG accumulation in three cases within the first 4–7 h. Gas was predominantly located within the hepatic veins, with no significant parenchymal, biliary, or arterial involvement, and only minor portal venous gas was noted in two cases, indicating a predominantly intravascular distribution. These findings are supported by Jackowski et al. [25] that likewise identified that the most frequent location of IHG was the hepatic venous system. However, Fischer et al. [38] further demonstrates that such intravascular gas also occurs in non-traumatic non-putrefied cases. Okumura et al. [10] demonstrated that focal or surface gas (both representing extravascular gas distributions), is highly specific for high-energy traumatic liver injury, whereas diffuse parenchymal gas is more consistent with decomposition. Overall, findings from the three studies [10, 25, 38] indicate that IHG is a non-specific finding with overlapping traumatic and non-traumatic causes, while extravascular gas patterns provide greater diagnostic specificity for trauma within an early PMI.

Egger et al. [43] documented that hepatic gas formation can occur independently of open wounds. In a preceding study of non-traumatic deaths without invasive medical intervention, PM gas formation was observed as early as five hours PM, although absent in some cases up to 84 h PM [40], indicating the variability of gas onset between individuals. Nevertheless, males showed higher prevalence of putrefactive gas that was attributed to longer PMIs, consistent with previous observations [45]. Importantly, results demonstrated that early PM gas was most commonly observed in the right heart cavities (~ 47%), followed by the hepatic parenchyma (~ 40%) [40]. The frequent cooccurrence of large gas volumes within both sites suggests a pathophysiological association [41], whereas gas detected in the left heart or periumbilical subcutaneous tissue was inconsistent with a putrefactive origin [40]. Because cardiac gas often appeared before hepatic gas during the early PM interval, distinguishing early PM gas from AM embolic gas may be particularly challenging [26, 29, 40, 50]. To improve discrimination, Egger et al. [40] proposed that gas grade III in the right heart cavity should prompt concurrent assessment of the hepatic parenchyma. In the absence of grade II hepatic parenchymal gas (moderate presence of gas, aggregated gas bubbles), the presence of cardiac gas grade III most likely originates from a non-putrefactive origin [40].

Building on these observations, Egger et al. [43] developed the RA-index, an objective scoring system to estimate the likelihood that PMCT gas is of a PM origin. Although several studies employed gas scoring systems, substantial heterogeneity was observed in their design and application. Most relied on binary or ordinal grading scales, while quantitative approaches with continuous data were rarely used. This limits direct comparison across studies [24] and may contribute to inconsistencies in reported thresholds for distinguishing gas origin. Nevertheless, the RA-index was the most frequently applied framework identified in this review (Table 5), providing a comparatively standardized approach to assessing gas distribution. The index combines weighted scores from seven anatomical sites to generate a composite score from 0 to 100, with scores > 50 supporting PM gas formation and lower scores in the presence of substantial intravascular gas raising suspicion of vital air embolism. However, even if scores are > 50, vital air embolism cannot be ruled out, as such events may precede PM gas formation. Validation studies on a sample size of 100 subjects demonstrated good reproducibility of the composite index despite only moderate agreement for individual site assessments.

The RA-index was developed from a heterogeneous cohort encompassing both traumatic and non-traumatic deaths, reducing the likelihood of bias from any single mechanism of gas formation. Subsequent studies found no seasonal variation in RA-index values despite faster gas development in warmer conditions [45] while others demonstrated a strong correlation between the RA-index and external decomposition scores [44]. Collectively, these findings reinforce the robustness of the RA-index as a tool for evaluating decomposition, although further validation across broader environmental and pathological contexts remains warranted, as cases involving decapitation or dismemberment, gas-producing infections, and putative effects of direct inhalation of exogenous inert gases, were excluded from the index [43].

The RA-index has been also been applied to differentiate gas patterns following intestinal perforation from putrefactive gas. In Hussein et al. [37], the absence of these early decomposition features, together with RA-index scores < 50 supported an AM gas origin, although authors noted that PMCT performed within one week PM has limited discriminatory capacity. Authors further suggested that contrast-enhanced CT imaging may aid localization of suspected perforations.

Beyond perforation, vascular collapse was observed in two-thirds of non-traumatic deaths with PMIs of 1–5 days when the RA-index was used to assess gas distribution [6]. While vascular collapse is traditionally associated with hemorrhage in trauma [51], its likelihood doubled when intravascular gas was also present, consistent with the known association that increases in intravascular gas accompany longer PMI [40, 44]. Vascular collapse on PMCT should therefore not be taken as specific for hemorrhagic shock, and requires cautious interpretation alongside trauma context and decomposition status. Importantly, no PMI threshold has been established beyond which PMCT gas findings lose diagnostic utility, and interindividual variability in gas formation is considerable, some cases show minimal gas despite prolonged PMI, while others accumulate substantial gas within hours of death.

Laurent et al. [19] considered gas findings following fatal diving incidents, comparing decompression illness (DCI) and non-decompression deaths (Table 7). Traditional DCI markers, including, pneumothorax, subcutaneous emphysema, and intraarterial gas demonstrated to be unspecific, whereas complete pneumatization of the supraaortic trunks and ventricles within 24 h PM was significantly associated with cerebral arterial gas embolism (CAGE). The reported percentage of individuals without CAGE (27.3%) combined decompression deaths with non-decompression dive deaths, which involve different mechanism of death, unrelated to DCI into a single no-CAGE population. However, this may be argued inaccurate, and when considering only decompression deaths, the rate of no-CAGE rises to 60%, suggesting that PMCT findings alone may have limited ability to distinguish between gas due to DCI and PM off-gassing.

Extending this line of investigation, Giaconi et al. [31] examined 32 cases with PMCT performed 24–48 h PM, including 10 scuba divers who died from AGE. They reported that intracorporeal gas in divers followed a distinct distribution pattern (significant amount of gas within the left heart chambers or supraaortic trunks) compared with fresh and putrefied corpses from unrelated deaths, suggesting that PMCT may retain diagnostic value for AGE even beyond the early PM period. While these findings imply potential utility beyond 24 h PM, the study did not fully account for PM off-gassing, and the accuracy of AGE diagnosis in the 10 diving cases remains uncertain. Current recommendations advocate performing PMCT within a narrower window, ideally within three hours PM [52], to minimize off-gassing effects, consistent with results from animal studies [53]. More broadly, timely PMCT acquisition appears important across forensic contexts where gas-related findings may be diagnostically relevant, as increasing PMI and progressive decomposition may substantially complicate interpretation of intravascular and intracorporeal gas distributions.

To address these limitations, Varlet et al. [20] focused on cardiac gas composition using headspace chromatography and thermal conductivity detection, in six scuba diving fatalities. They also introduced the RA-index score, which did not correlate with decomposition state, indicating that PMCT findings are most informative when interpreted alongside compositional analysis rather than relying on a scoring index alone. Notably, in cases with short PM intervals, cardiac CO₂ concentration may provide additional insight into cause of death when considered in the context of the diving profile. While promising, this approach requires validation in larger prospective studies to confirm its utility in diagnosing diving-related deaths.

It should be noted that CPR procedures with chest compressions [29], endotracheal positive ventilation and venous catheters [27, 34] need to be considered during PMCT gas interpretations. Likewise, it is well established that in penetrating trauma, such as stabbing or gunshot wounds, gas accumulations may delineate wound tracts [47, 54]. Pneumothorax represents a further consideration in trauma cases. Lopatin et al. [32] demonstrated a significant correlation between pneumothorax detected on PMCT and thoracic injuries in a large trauma cohort, underscoring that its presence in non-diving deaths is more reliably attributable to mechanical injury than to decompression-related or CPR-related mechanisms, though the two cannot always be distinguished without contextual information. Intracranial gas warrants particular interpretive caution. Smith et al. [42] characterized PM brain CT findings within 72 h and identified several pitfalls in interpretation, noting that gas within the intracranial compartment may arise from PM changes rather than reflecting AM pathology, such as traumatic pneumocephalus or cerebral gas embolism. These observations are consistent with findings from Borowska-Solonynko et al. [33], who demonstrated that intracranial gas on PMCT in traumatic deaths requires careful contextualization to avoid misattribution of origin. In less obvious traumatic cases, small solitary bubbles may improve diagnostic accuracy as they are associated with the VP. Referred to as minute gas, VP is well documented in clinical radiology, most frequently associated with degenerative joint disease though has also been reported in abscesses, osteomyelitis and malignancies (including multiple myeloma and metastasis), as well as traumatic lesions such as fractures, intervertebral separation and ligament tears [35, 36, 55].

The VP is thought to arise through a pressure-dependent process, where expansion of an enclosed space, such as a joint, reduces gas solubility in surrounding tissues (Boyle’s and Henry’s laws), allowing gas to come out of solution [55]. The VP has traditionally been observed in living individuals at sites of trauma, particularly near rib fractures and joint spaces [56], suggesting a potential link to mechanical forces rather than PM changes.

PMCT studies have investigated the VP in different regions to assess its forensic relevance. Schulze et al. [46] described a “gas bubble sign” adjacent to laryngeal fractures, originally reporting a positive predictive value of 95%, overall accuracy of 83%, sensitivity of 79%, and specificity of 91%. Their assignment of true and false positives and negatives were not defined according to standard diagnostic testing and when interpreted according to standard conventions, sensitivity increases to 95% while specificity decreases to 67%. The high sensitivity suggests that the gas bubble sign may reliably detect fractures when present, but the lower specificity indicates that gas can also appear in the absence of fractures. Given the small study population, these metrics should be interpreted cautiously, as estimates may not generalize to larger cohorts. Similarly, Lyness et al. [16] observed that gas bubbles near laryngeal structures were often present without fractures, highlighting that gas alone is not a consistently reliable indicator of trauma.

Chatzaraki et al. [35] found that central VPs in the thoracic and lumbar intervertebral disc correlated with hangings in younger individuals, whereas peripheral VPs were more common in controls without trauma. In contrast, Kudo et al. [36] reported that centrally located gas within the cervical intervertebral discs reflected degenerative changes rather than trauma, indicating that interpretation depends on anatomical location and underlying pathology.

Overall, while VP may reflect AM mechanical events such as trauma, its presence is not a definitive diagnostic marker. Sensitivity and specificity vary by region and study, and methodological limitations, including small sample sizes, inconsistent definitions, and potential PM confounders, reduce confidence in its forensic utility. Consequently, VP should be interpreted cautiously, ideally in conjunction with other evidence, and further research is needed to clarify whether it can reliably distinguish gas origin.

Although not yet part of routine casework, gas composition analysis has proven to be a valuable complement to PMCT gas assessment [30]. CT-guided headspace chromatography of cardiac gases from cases with varying causes of death and PMI has shown that hydrogen (H₂), hydrogen sulfide (H₂S), and methane (CH₄) are indicative of decomposition, whereas a CO₂/N₂ ratio < 0.2 suggests gas embolism and > 0.2 indicates decomposition [30]. When integrated with PMCT findings and the RA-index, diagnostic accuracy improves: embolic cases exhibit elevated CO₂ (20–50%), CO₂/N₂ >0.2, and RA-index < 50, while traumatic and decomposition cases show lower ratios (< 0.2). Together, gas composition analysis and PMCT may provide a complementary approach for supporting differentiation between vital embolism, passive gas entry, and decomposition. Interpretation of PMCT gas findings therefore requires consideration of the entire forensic context, including PMI, decomposition status, trauma pattern, resuscitative interventions, environmental conditions, and mechanism of death.

### Study limitations

This review has several limitations arising from the methodological constraints inherent to scoping reviews [57]. First, a potential selection bias exists, as only 28 studies met the eligibility criteria, which should be considered when interpreting the findings. Moreover, the review was restricted to human studies published in English-language, peer-reviewed journals, and only original research articles were included. Consequently, studies in other languages, animal studies, and data reported in case studies or doctoral theses were excluded. Institutional data from centers routinely performing PMCT that have not been published in peer-reviewed journals were not considered.

As reported in our results, 79% of studies were retrospective, with an almost equal share being descriptive or correlative, with no experimental or causal-effect studies identified. These inherent design constraints, combined with the practical limitations of forensic death investigations, such as the inability to implement controlled crossover studies or stratified populations restrict the availability and quality of evidence. Consequently, much of the current understanding of PMCT findings relies on observational data, which limits the ability to draw definitive conclusions about causality or the effects of specific variables such as helium exposure or septicemia. 

### Implications

- Extravascular hepatic gas seen on PMCT within the first 24 h PM is most consistent with systemic or traumatic mechanisms, although gas of putrefactive origin cannot be excluded within this timespan.
- Gas in the cardiac cavities observed within the first 24 h PM most often reflects systemic or traumatic causes. If both cardiac gas and IHP gas are noted simultaneously, gas is more likely putrefactive in origin.
- Gas confined to the cardiac cavities within 24 h PM may reflect CPR-related artefact, although in certain traumatic settings it may also indicate a vital pathological process.
- Presence of gas in vessels increases the risk of vessel collapse and possibly misinterpretation as vessel collapse due to major blood loss.
- In diving accidents, effects from off-gassing and decomposition make diagnosis of AM embolism difficult. PMCT images should be interpreted alongside the dive profile and gas sampling with compositional analysis may be considered in order to enhance diagnostic accuracy.
- Small gas bubbles near laryngeal structures may correlate with neck trauma or laryngeal fracture though a causal association has not been established.

## Conclusions

Gas analysis PM is challenging, as the topic lies at the intersection of known and assumed pathophysiological mechanisms, and concurrently balances between normal and abnormal morphology. Nonetheless, certain early distribution patterns can aid interpretation by applying the RA-index as well as the cardiac-hepatic diagnostic rule both developed by Egger and colleagues. Combined assessment of cardiac and hepatic gas distribution may aid differentiation between early PM gas formation and AM embolic gas, with marked cardiac gas in the absence of moderate hepatic parenchymal gas suggesting a non-putrefactive origin. Distribution patterns surrounding the liver indicate that extravascular focal gas favored high-energy traumatic injury, while diffuse parenchymal gas is more consistent with decomposition. In diving fatalities, off-gassing significantly reduces limits the ability to diagnose AM embolism using PMCT and gas sampling should be considered to improve accuracy. Small gas bubbles near the laryngeal structures, when other sources and decomposition are excluded, may indicate trauma. Conclusively, interpreting PMCT gas distribution patterns with contextual findings and supplementary analyses may suggest a probable mechanism, though further research is warranted.

## Data Availability

Not applicable. Data is based on published studies.
